# Perimenopausal women show modulation of excitatory and inhibitory neuromuscular mechanisms

**DOI:** 10.1186/s12905-021-01275-8

**Published:** 2021-03-31

**Authors:** Heidi Pesonen, Eija K. Laakkonen, Pekka Hautasaari, Pauliina Aukee, Vuokko Kovanen, Sarianna Sipilä, Taija Finni, Ina M. Tarkka

**Affiliations:** 1 Faculty of Sport and Health Sciences, University of Jyväskylä, P.O. Box 35, 40014 Jyväskylä, Finland; 2 Gerontology Research Center, University of Jyväskylä, Jyväskylä, Finland; 3 Department of Obstetrics and Gynecology, Pelvic Floor Research and Therapy Unit, Central Finland Central Hospital, Jyväskylä, Finland

**Keywords:** Menopause, Follicle-stimulating hormone, Motor cortex, TMS silent period, Twitch force potentiation

## Abstract

**Background:**

Menopausal transition exposes women to an early decline in muscle force and motor function. Changes in muscle quality and function, especially in lower limbs, are crucial, as they expose individuals to increased risk of falls. To elucidate some of the related neuromuscular mechanisms, we investigated cortical inhibition and peripheral muscle twitch force potentiation in women during the early and late stages of perimenopause.

**Methods:**

Participants were 63 women aged 48–55 years categorized as early (EP, n = 25) or late (LP, n = 38) perimenopausal according to serum follicle-stimulating hormone (FSH) levels and menstrual diaries. EP women had an irregular menstrual cycle and FSH < 25 IU/L, while LP women had an irregular cycle and > 25 IU/L. We examined motor evoked potential (MEP) and silent period (SP) elicited by transcranial magnetic stimulation (TMS), in the tibialis anterior muscle at 20%, 40%, and 60% of maximal voluntary contraction (MVC) levels, and twitch force potentiation in plantar flexors.

**Results:**

EP group showed a longer SP duration in 40% MVC condition and larger motor evoked potential amplitude in 20% MVC condition compared to the LP group. No group difference was detected in twitch force potentiation; however, it correlated negatively with FSH levels. Other factors, such as age, height, body mass index, or physical activity did not explain group differences.

**Conclusions:**

Our preliminary results indicate subtle modulation in both TMS-induced inhibitory and excitatory mechanisms and twitch force potentiation in women already in the late perimenopausal stage. This suggests that the reduction of estrogens may have an accelerating role in the aging process of neuromuscular control.

## Background

Menopausal transition, i.e. perimenopause, is characterized by a range of physiological changes caused by alterations in female hormone levels [1]. These changes expose women to an early decline in muscle quality and motor function [2, 3]. Together with changes in muscular properties, neurophysiological and cortical mechanisms play a major role in motor deficits related to the normal aging process [4, 5]. As changes in muscle function and motor control especially in lower limbs expose individuals to increased risk of falls [6, 7], understanding alterations both in muscle function and cortical control is increasingly important. Female sex hormones have a strong neuroprotective role and reduction of estrogens during menopausal transition is associated with multiple neurophysiological changes, such as neuroinflammation, mitochondrial dysfunction, and synaptic decline [8, 9]. While menopause is recognized as a reproductive transition, yet a variety of neural changes are known to occur, and several perimenopausal symptoms are mainly neurological [1].

Transcranial magnetic stimulation (TMS) is a pain-free, non-invasive clinical and therapeutic tool widely used in research to study cortical excitatory and inhibitory mechanisms [10–13]. TMS-elicited silent period (SP) is used to study cortical inhibition and is detected as suppression of on-going activity in surface electromyogram (EMG) of a contracted muscle, following TMS-elicited motor evoked potential (MEP) [14, 15]. The physiology of SP is under debate, but the early part of the silent period is suggested to originate from spinal mechanisms and the later part from cortical and possibly overlapping spinal inhibitory mechanisms [12, 16]. SP is believed to represent gamma-aminobutyric acid (GABA) receptor-mediated inhibitory mechanisms [12]. Pharmacological studies suggest this inhibition arises mostly through type B receptors (GABA_B_) [18, 19]. Despite inter-individual variability in SP durations, consistent SP modulation is found in various clinical conditions, such as multiple sclerosis and Parkinson’s disease [20, 21].

GABAergic levels are shown to decrease in aging and possibly even more pronounced in aging women [22, 23]. Female sex hormones are known to influence excitatory and inhibitory mechanisms including the GABAergic system [24]. TMS-induced inhibitory mechanisms have been studied in the normal aging process. Aging effects on SP are not entirely clear, however, both a decrease and no change in SP duration have been found in older adults compared to young [4, 25, 26]. Another TMS technique, paired-pulse TMS, has revealed both lengthening and shortening in intracortical inhibition, believed to represent GABA_A_ergic inhibition [27, 28]. Inhibitory and excitatory neuromuscular mechanisms have not been studied during the menopausal transition, despite the changes in muscle function and force production. As menopausal transition seems to accelerate the changes observed during the normal aging process, corticospinal inhibitory mechanisms may demonstrate this modulation early on and thus are of interest in the study of menopause.

Force potentiation is a phenomenon in skeletal muscles, where produced force is temporarily enhanced by recent muscle activity [29, 30]. Twitch force potentiation is induced by peripheral electrical stimulation before and after conditioning voluntary muscle contraction. The mechanism behind muscle twitch force potentiation is believed to be phosphorylation of myosin regulatory light chains (pRLC), which makes actin and myosin more sensitive to Ca^2+^ and alters the structure of the myosin head [31]. Another suggested mechanism for force potentiation is the increase in recruitment of higher-order motor units [29]. Twitch force potentiation is shown to be lower in older adults and, interestingly, decreases have been observed already in women aged 45–54 years, about the same age as menopause [32–34]. Estradiol is previously connected to modulation of force potentiation mechanisms and myosin pRLC in mice [35]. Furthermore, Finni et al. [36] found higher twitch torque in postmenopausal women who were users of estrogen-containing hormone replacement therapy (HRT) compared to their monozygotic co-twins, who had never used HRT, without a difference in voluntary force generation. This suggests that modulation of involuntary force generation may be an initial indicator of decline in muscle force in postmenopausal women.

Menopause exposes women to an early decline in physical function and muscle force [2, 3]. The ability to perform coordinated movements in normal daily living relies on inhibitory and excitatory control. So far, neuromuscular mechanisms have not been investigated in menopausal women. In the present study, we investigated if both TMS-induced SP and peripheral twitch force potentiation are modulated in early and late perimenopausal women, with the changes in hormonal levels already present. The authors hypothesized a modulation in TMS-induced corticospinal inhibitory mechanisms, possibly seen as a shortening of SP, due to changes earlier observed in the aging population [25, 26]. As earlier research shows the modulation in twitch torque in postmenopausal women and modulation of pRLC due to changes in estradiol levels, we also hypothesized a decrease in twitch force potentiation [35, 36]. We recorded neuromuscular data and lower limb functional abilities from 63 women in the early and late stages of perimenopause. We chose lower limbs as our target because of their important role in functional abilities and balance maintenance during the aging process.

## Methods

### Study protocol

The participants were 63 women aged 48–55 (mean 51.4) years, a representative subgroup of the study population of the Estrogen Regulation of Muscle Apoptosis (ERMA) –project organized at the Gerontology Research Center (GEREC) and the Faculty of Sport and Health Sciences at University of Jyväskylä [37]. The complete ERMA-study protocol, including the current sub-study, was approved by the ethics committee of the Central Finland Health Care District (K-SSHP Dnro 8U/2014), Jyväskylä, Finland. The initial study population was randomly selected from the Finnish National Registry, kept by the Population Register Centre, targeting women aged 47 to 55 years living in the Jyväskylä area. An invitation letter with a prequestionnaire and general consent was sent to 6,878 women, from whom 46.9% responded. A total of 1,627 participants, fitting the inclusion criteria and consented, were invited to the laboratory visit. During the visit, a structured health interview was assessed, fasting blood samples were collected, and participants filled an informed consent for the subsequent phases of the ERMA study. Participants also kept a menstrual diary for at least 12 weeks. Exclusion criteria included estrogen-containing hormonal preparations or other medications affecting ovarian function, current pregnancy or lactation, conditions affecting ovarian function, including bilateral oophorectomy, body mass index (BMI) > 35 kg/m^2^ (based on self-reported height and weight), and chronic diseases or medications seriously affecting muscle function. If a participant reported serious or unclear health problems, they were examined by a physician to ensure safe participation in physical performance tests.

The core-ERMA group used in the current study consists of women who had natural reproductive status, i.e., they had an intact uterus and they had not used during the past three months or were not currently using any hormonal contraception or other medication that could affect following up their menstrual bleeding pattern [37]. Menopausal status was determined by measuring participant’s follicle-stimulating hormone (FSH) level from a blood sample taken, if possible, during the first five days of the menstrual cycle and by recordings in the menstrual calendar kept for six to twelve months. Also, each participant’s 17β-estradiol (E2) levels were measured. FSH and E2 were detected with immunoassay using IMMULITE 2000 XPi (Siemens Healthcare Diagnostics, UK). All participants who took part in the current study with electrophysiological measurements were perimenopausal, without hormonal contraception, and with an intact uterus. Their perimenopausal status was further defined following Stages of Reproductive Aging Workshop guidelines [38] defined as *early perimenopausal (EP)* if FSH was below 25 IU/L and irregular menstrual cycle was reported and *late perimenopausal (LP)* if FSH was over 25 IU/L. Women with FSH over 30 IU/L and no menstrual bleeding during the past three to six months were considered postmenopausal and thus they were excluded from the current study. In addition, women with FSH below 17 IU/L and reporting regular menses were considered premenopausal and thus they also were excluded from the current study. The first three months of menstrual cycle length, the number of bleeding days, and the number of non-bleeding days were calculated for each participant. Three participants from the EP group and four from the LP group did not report any bleeding days during this time.

**Fig. 1:**
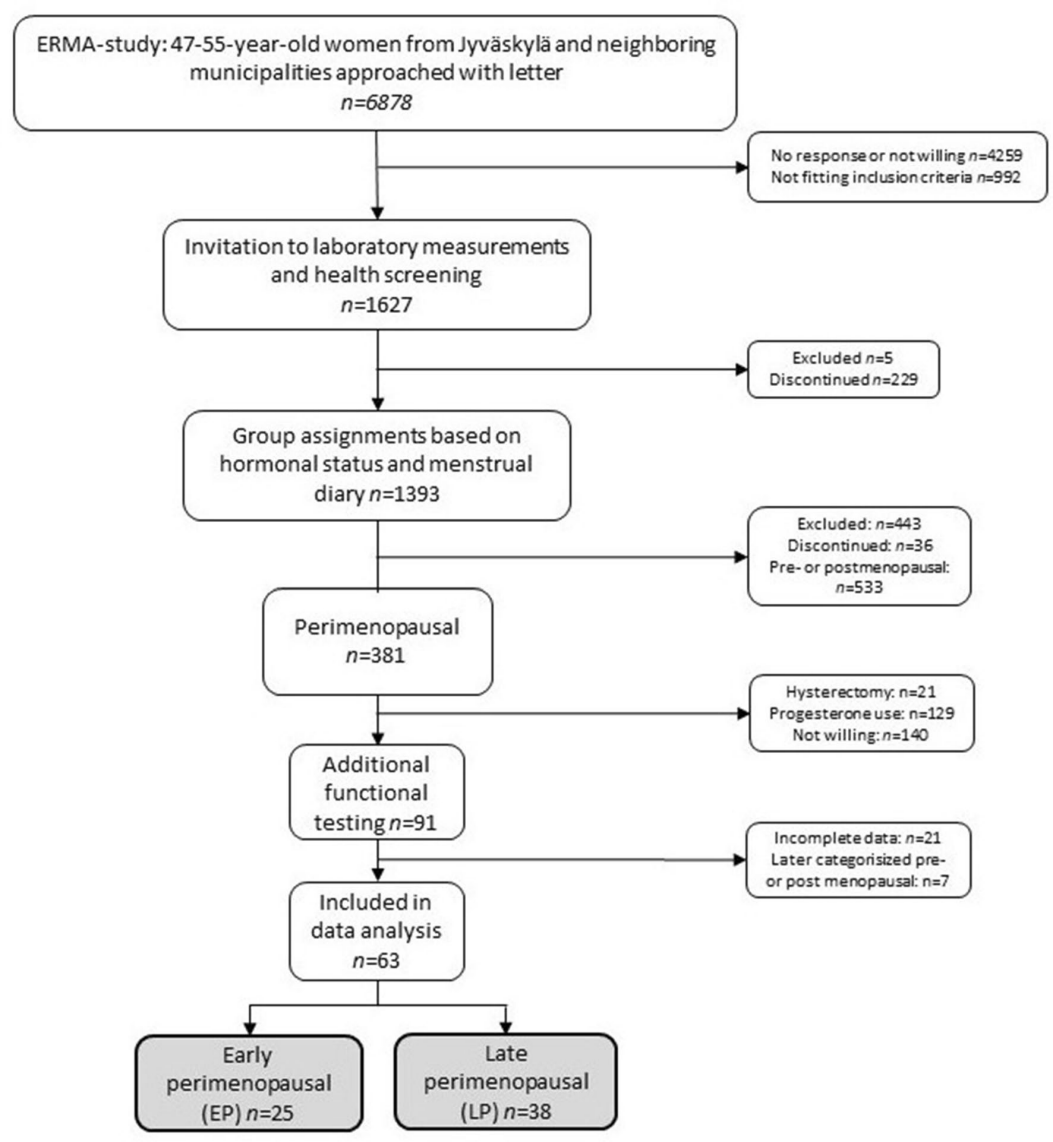
Flow chart of the recruitment process of the study population

Women defined to be perimenopausal with a natural hormonal cycle were invited to take part in a subset of functional tests. Ninety-one women volunteered. Researchers performing the measurements and data analysis were blinded to participant’s menopausal status and other background information. Good quality electrophysiological data were obtained from 63 participants, from which 25 were EP and 38 were LP (Fig. 1).

### Functional measurements

*Physical activity (PA) level* was determined by the seven-point scale for the current level of weekly leisure-time PA [39]. The scale was previously shown to correlate well with accelerometer-based PA and mobility variables [40]. Questionnaire response categories were: (0) inactive, (1) light activity 1 to 2 times per week, (2) light activity several times per week, (3) moderate activity 1 to 2 times per week, (4) moderate activity several times per week, (5) high activity several times per week, and (6) competitive sports and related training several times per week. Categories 0 and 1 were further combined to low activity group, 2 and 3 to moderate activity group, and 4, 5, and 6 to high activity group.

*Maximal isometric knee extension* strength was measured in Newton-meters (Nm) in one leg, dominant hand side, with a Good Strength -dynamometer chair (Metitur Oy, Jyväskylä, Finland). The participant’s knee was set at 60° angle from full extension and the ankle was strapped to force transducer. The participant was instructed to extend the knee with maximal force with verbal encouragement. The best performance of three to five isometric extensions was selected for the analysis.

*Vertical jumping height* was measured three to five times utilizing a contact mat. The jumping height indicates the participant’s ability to elevate the body’s center of gravity during a vertical countermovement jump. Flight time (t) was measured, and vertical jumping height (m) was calculated: (g × t^2^) / 8 × 100^25^. The highest value was selected for the analysis.

*Electrophysiological measurements* were performed on the participants’ right leg, while she sat in an ankle dynamometer chair, custom made in the University of Jyväskylä [41], with her back and head resting against the backrest. The knee was extended at 180 º and the foot was strapped against the footplate at a 90 º angle. Two surface electrodes (Ambu® BlueSensor N, 22 × 28 mm, Ballerup, DK) were placed on tibialis anterior (TA) and medial gastrocnemius (MG) muscles for bipolar EMG recording, with a 20 mm interelectrode distance and a ground electrode placed proximally.

Before TMS procedures, maximal voluntary contraction (MVC) torque for ankle dorsiflexion was determined. MVC was defined as the highest torque from two maximal dorsiflexion contractions and 20%, 40%, and 60% of MVC were calculated. TMS was performed with Magstim Rapid^2^ stimulator (Magstim, Whitland, UK) using a double 70 mm coil (figure-of-eight coil). Single-pulse stimulation was applied on the motor cortex over the measured and marked center of the scalp, with a 45° angle from the mid-sagittal lane to target the lower limb representation area on the contralateral hemisphere. Stimulation intensity was increased gradually to find the “hotspot” and assess the resting motor threshold (RMT). Individual RMT in the lower limb was set to the occurrence of 50 µV MEP in relaxed TA consecutively three times. Since the lower limb motor cortex locates deep in the central sulcus and may need relatively strong stimulus intensity to elicit MEP, we recorded the used stimulator output for each participant to control for its possible effects. This individual stimulation intensity was used while the target muscle was contracted. Participants were instructed to perform isometric dorsiflexion at 20%, 40%, and 60% of their MVC, with rest periods in between. Such submaximal force levels are recommended e.g. by Säisänen et al. [14], as they are relatively easy to maintain during measurement. Produced force and the target force level were shown on the screen in front of the participants. The contraction was held continuously, while 6 to 10 stimulations were delivered at the individual hotspot with 10-s inter-stimulus intervals.

For twitch force recording, peripheral electrical stimulation was delivered to the tibial nerve in the popliteal fossa. The optimal stimulation point was located while the participant was lying prone. Gradually increasing stimulation intensity optimal location and intensity were defined where the peak-to-peak amplitude of the M-wave in the MG muscle and the configuration of the M-wave was repeatable for a minimum of three times. The cathode (Ambu® WhiteSensor 4500 M, 79 mm^2^, Ballerup, DK) electrode was placed in this location and the anode electrode (V-trodes; Mettler Electronics, Anaheim, CA, USA) was placed slightly proximal to the patellofemoral joint. Supramaximal electrical stimulation of 1 ms duration, with 150% intensity of individual maximal M-wave, was delivered with a constant current stimulator (DS7AH, Digitimer Ltd., Hertfordshire, UK). The participant was then seated in the dynamometer chair and instructed to relax, while the first stimulus was delivered, and after that to perform a maximal isometric plantarflexion, during which the second stimulus was delivered. After 2–5 s relaxation, the third stimulus was delivered to a relaxed muscle. Participants received verbal encouragement to perform the contraction and visual feedback of their torque level from the screen. Three trials including three stimulations were performed with 60-s rest intervals in between.

EMG signals were amplified 1000 × and band-pass filtered (10 Hz–1 kHz). The ankle MVC was measured with a torque-transducer (Kistler Group, Switzerland), mounted between the ergometer servomotor and the platform of the foot.

### Data analysis

For TMS data, individual MEP and SP responses were analyzed manually (Spike2 version 6.17, Cambridge Electronics Design, Cambridge, UK). MEP-start was detected as the point where the TA EMG signal exceeded baseline activity and the MEP-end as the point where the complete EMG silence began. This same time-point was also the SP-start. MEP amplitude was determined as the largest peak-to-peak amplitude. SP-end was where baseline EMG activity returned. For statistical analysis, both “absolute” SP duration (SP-start – SP-end) and “relative” SP (MEP-start – SP-end) were recorded [14, 42]. If a participant had four or more successful MEPs and SPs in one force level, those with the shortest and longest durations were not included in further analysis. From the remaining recordings, mean values for SP and MEP for each participant were calculated for each force level.

**Fig. 2:**
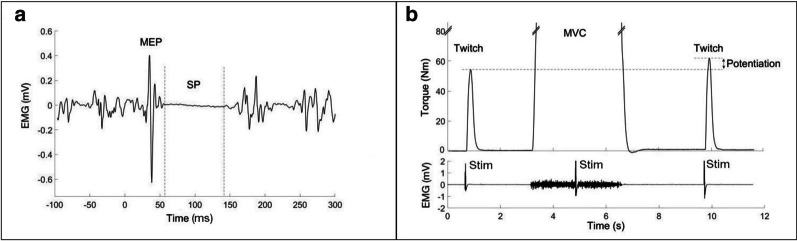
A. EMG recording of TMS response from a single participant at 40% MVC condition in tibialis anterior muscle. Dashed lines indicate the start and end of SP. MEP amplitude was calculated as the maximum peak-to-peak amplitude. B. Torque data of twitch force potentiation analysis is presented from a single participant. Potentiation was calculated as the difference between pre-MVC twitch and post-MVC twitch. Stimulus artifacts are detectable in the EMG recording from the medial gastrocnemius muscle

The electrical stimulation data were analyzed using Matlab (R2015a, 8.5.0, Mathworks Inc., Natick, MA, USA). Peak twitch torque (PTT) and maximal voluntary contraction (MVC) torque were manually detected from the torque signal (Fig. 2). Twitch force potentiation was analyzed by comparing PTT of pre-MVC twitch peak amplitude to post-MVC twitch peak amplitude in the recorded three trials using the equation: twitch potentiation (%) = ((post-MVC PTT/pre-MVC PTT)/pre-MVC PTT) × 100. The best potentiation effect and the best result from MVC out of three trials were selected for further analysis.

Statistical analysis was performed with IBM SPSS Statistics Version 24 (IBM Corporation, Chicago, IL, USA). Group comparisons between EP and LP women were performed with independent samples t-tests and for PA variable with chi-square test. Variability between different stimulation conditions was tested with paired-sample t-test. Correlations were detected with Pearson correlation coefficient or ANOVA. Covariance was tested with ANCOVA. The significance level was set to p < 0.05.

## Results

### Group characteristics

Both groups were approximately the same age, height, and BMI (see Table 1). As expected, the EP group had lower FSH levels and higher E2 levels than the LP group as well as a shorter duration of the menstrual cycle for the first three months of the follow-up period. No differences were detected in PA habits between groups. Stimulus intensities varied across participants but there were no differences between groups.

**Table 1:** Characteristics of the participants, mean (± SD) or n (%)

	EP (n = 25)	LP (n = 38)	P-value
Age, y	51.0 (± 2.0)	51.6 (± 1.8)	0.222
Height, cm	163.8 (± 0.1)	164.2 (± 0.1)	0.810
BMI	25.4 (± 4.4)	25.3 (± 3.7)	0.916
FSH (IU/L)	17.32 (± 4.79)	48.25 (± 22.28)	< 0.001
E2 (nmol/L)	0.374 (± 0.257)	0.249 (± 0.200)	0.035
Mean cycle length	36.8 (± 22.2) ^a^	65.8 (± 41.7)^b^	0.001
Mean bleeding days	6.0 (± 1.4) ^a^	5.3 (± 1.5)^b^	0.098
Mean non-bleeding days	34.7 (± 31.6) ^a^	65.2 (± 49.1)^b^	0.006
Physical activity			0.652
Low	4 (16)	6 (16)	
Moderate	7 (28)	7 (18)	
High	14 (56)	25 (66)	
Stimulus intensity (%)	83.6 (± 7.7)	84.5 (± 9,6)	0.691

P-values tested with independent samples t-test, physical activity level with chi square test. BMI = body mass index, cm = centimeter, E2 = 17β-estradiol, FSH = follicle-stimulating hormone, IU/L = international units per liter, nmol/L = nanomoles per liter, SD = standard deviation, y = year. ^a^n = 23, ^b^n = 34.

### Silent period

**Fig. 3:**
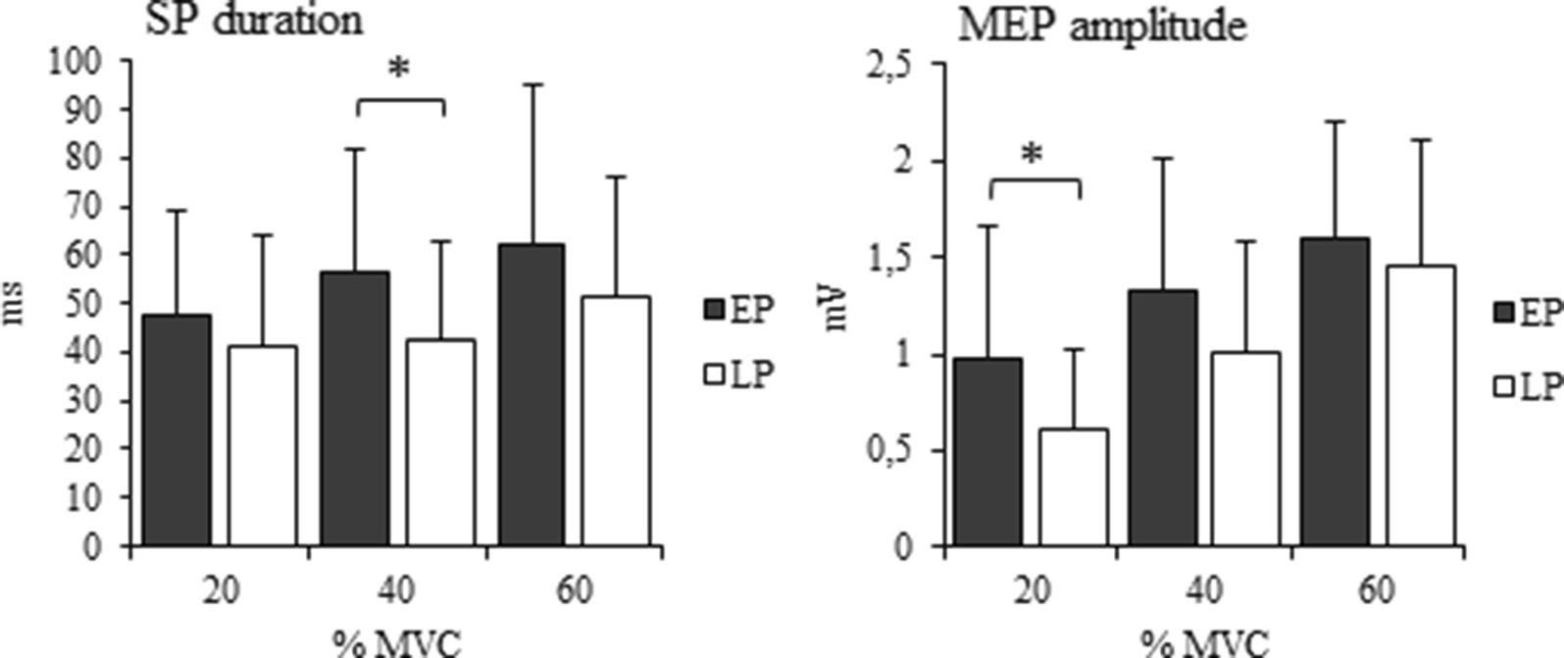
Mean SP duration and MEP amplitude (ms and mV, error bar indicates SD) in EP (n = 25) and LP (n = 38) groups, recorded in all three (20%, 40% and 60% MVC) conditions. * indicates significant difference (p < .05)

SP duration differed between EP and LP groups in 40% MVC condition (t(61) = 2.473, p = 0.016). Mean absolute SP duration for the EP group was 57.1 ± 25.8 ms and for the LP group 42.8 ± 20.1 ms. The difference remained after controlling for stimulus intensity (F(1,60) = 6.044, p = 0.017). There was no significant difference between groups in 20% MVC (47.8 ± 22.0/41.1 ± 22.7, t(61) = 1.156, p = 0.252) and 60% MVC (62.2 ± 33.6/51.3 ± 24.5, t(61) = 1.491, p = 0.141) conditions (Fig. 3). Duration of SP increased with required muscle force for all participants (p < 0.01). In EP group the increase in SP duration was significant from 20 to 40% MVC conditions (p < 0.01) and in LP group from 40 to 60% MVC conditions (p < 0.01) (Fig. 3).

When analyzing relative SP, it also differed between groups in 40% MVC condition (t(61) = 2.494, p = 0.015). Mean relative SP duration for EP group was 88.4 ± 29.4 ms and for LP group 72.1 ± 22.5 ms. No significant difference was found in 20% MVC (78.4 ± 25.6 ms/68.7 ± 25.5 ms, t(61) = 1.475, p = 0.145) or 60% MVC (93.2 ± 38.1 ms/80.9 ± 26.9 ms, t(61) = 1.507, p = 0.137) conditions.

### Motor evoked potential

MEP amplitude differed between groups in 20% MVC condition (t(61) = 2.511, p = 0.017). EP group mean amplitude was 1.000 ± 0.688 mV and for LP group 0.617 ± 0.409 mV. The difference remained when controlling for stimulus intensity (F(1,60) = 7.460, p = 0.008). MEP amplitudes showed a similar tendency in 40% MVC condition, as mean amplitude was 1.326 ± 0.707 mV in EP group and 1.010 ± 0.576 mV in LP group (t(61) = 1.944, p = 0.057), albeit not significant. There was no significant difference in MEP amplitudes in 60% MVC condition (1.598 ± 0.607 mV/1.454 ± 0.648 mV, t(62) = 0.883, p = 0.381). MEP amplitude increased with muscle force in both groups (p < 0.001).

**Fig. 4:**
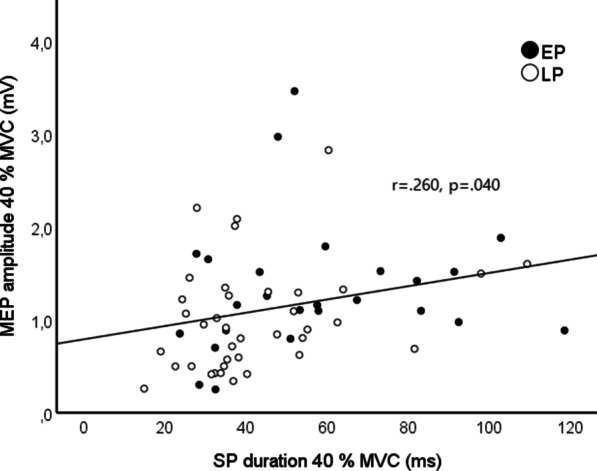
Association between mean MEP amplitude (mV) and SP duration (ms) in 40% MVC condition. Black circles represent the EP group (n = 25) and white circles the LP group (n = 38)

Absolute SP duration and MEP amplitude correlated in 40% MVC condition (r = 0.260, p = 0.040) (Fig. 4) and 20% MVC condition showed similar tendency, but not significant (r = 0.235, p = 0.063). No correlation was found between SP duration and MEP amplitude in 60% MVC condition (r = 0.077, p = 0.548).

### Twitch force potentiation and physical performance

Twitch force potentiation did not differ between groups. An association between twitch force potentiation and FSH (r = -0.262, p = 0.043) was observed. No association was found between twitch force potentiation and E2 (r = 0.123, p = 0.348). There were no differences between groups in vertical jumping height, knee extension strength, or ankle dorsiflexion strength (Table 2). However, EP group had 17.2% stronger plantar flexion compared to LP group (t(57) = 2.533, p = 0.014). There was no correlation between plantar flexion torque and SP duration in 40% MVC condition (r = 0.029, p = 0.830) nor with plantarflexion torque and MEP amplitude in 20% condition (r = 0.130, p = 0.327).

**Table 2:** Measures of lower limb physical performance and twitch force potentiation, mean (± SD)

	EP (n = 25)	LP (n = 38)	P-value
MVC dorsiflexion (Nm)	31.9 (± 9.1)	34.3 (± 6.7)	0.238
MVC plantarflexion (Nm)	131.1 (± 33.9)^b^	108.8 (± 32.4)^b^	0.014
Knee extension strength (Nm)	162.2 (± 27.9)^b^	153.7 (± 38.8)^c^	0.360
Vertical jumping height (m)	0.185 (± 0.046)^a^	0.200 (± 0.037)^a^	0.177
Twitch force potentiation (%)	10.1 (± 6.3)^a^	6.7 (± 9.3)^b^	0.124

P-values tested with independent samples t-test. MVC = maximal voluntary contraction, m = meter, Nm = Newton meter, SD = standard deviation. ^a^n = missing one, ^b^n = missing two, ^c^n = missing five.

## Discussion

The aim of the present study was to examine TMS-induced inhibition and peripheral twitch force potentiation in women in early and late perimenopausal stages, to determine if the progression of the menopausal stage affects motor control mechanisms. Our finding was that women in the EP group presented longer SP durations than women in the LP during a moderate force production condition (40% MVC). MEP amplitudes were sensitive to the perimenopausal stage as in 20% MVC condition EP group showed larger MEP amplitudes and the same tendency remained in 40% MVC condition. Furthermore, an association was observed between twitch force potentiation and FSH. Our participants were comparable in age, height, BMI, and PA level and thus those factors do not explain the differences we detected.

Our results indicate a reduction in corticospinal inhibitory mechanisms observable already in late perimenopause. While pharmacological studies have provided strong evidence for GABAergic origins of SP [17–19, 43], there remains a debate over the spinal and cortical contribution to the generation of SP. Formerly, the early part (~ 50 ms) of SP has been thought to arise from spinal origins and the later part from cortical origins [16–19, 42, 44]. Recently Yacyshyn et al. [45] suggested that spinal inhibitory mechanisms could play an even larger role in SP generation than previously thought. They found cervicomedullary motor evoked potential suppression at up to 150 ms after TMS. However, as long-lasting cortical GABAergic neurons probably affect SP throughout its duration, it is unclear how the spinal and cortical inhibitory mechanisms interact in the latter part of the SP. Further studies are needed to determine their roles in SP generation.

During aging, competencies indicating good motor coordination and performance are important, as they reflect an individual’s overall motor abilities and the risk of falls [6, 7]. This association is further emphasized in aging women, as menopause exposes them to early decline of muscle function [2, 3]. TMS studies indicate a strong cortical contribution in controlling muscle activity e.g. during the gait cycle, that can be modulated by intracortical inhibition [46]. There is also growing evidence that older individuals utilize somewhat different cortical mechanisms in motor control tasks compared to young [47, 48]. Along with decreasing levels of GABA [22, 23], decreased inhibition has been observed in older adults, and it has been connected to decreased control of coordinated movements [25, 26, 49, 50]. Recently, Swanson and Fling [50, 51] reported shorter SP in older adults than young. Older adults also demonstrated an association between shorter SP and reduced performance in walking and turning, while an opposite correlation was observed for young adults. This was only present in the right hemisphere stimulation. The results suggest that decreased inhibitory control may underlie age-related decline in motor control and coordination. Our results offer a proposal that changes in hormonal levels in menopausal transition may accelerate the decrease in inhibitory mechanisms in cortical or spinal levels and possibly accelerate the aging process of motor function. Unlike the study by Swanson & Fling [50], we performed the stimulation only to the left hemisphere, where they did not find significant differences between young and older adults. Therefore, we cannot make further conclusions about hemispheric differences.

Furthermore, we found lower MEP amplitude in LP than in the EP group indicating modulation of cortical excitability in perimenopausal stages. Reduced MEP amplitudes in older adults compared to young are a common finding, and our results suggest that menopause may accelerate this modulation [25, 26, 49]. Here, SP durations and MEP amplitudes were associated in moderate force production condition. This relationship between SP and MEP has been previously reported [14, 25, 52]. Therefore, the mechanisms that underlie the changes observed in SP and MEP, may have similar origins.

Twitch force potentiation did not differ between EP and LP groups. However, there was a negative correlation between participants’ FSH level and twitch force potentiation. This correlation suggests modulation toward decreasing twitch force potentiation in the menopausal transition. FSH level fluctuates also during menstrual cycle in women. However, no association has been found earlier with involuntary muscle contractile properties and FSH levels during menstrual cycle [53]. A correlation was not present with E2 but only with FHS, which may be more sensitive here to different stages of perimenopause. Furthermore, the very subtle fluctuations of E2 may not be detected with IMMULITE 2000 XPi. Twitch force potentiation and twitch torque have been shown to decrease in aging women and are suggested to partly underlie the morphological and functional changes observed in aged muscle [33, 36]. As estradiol seems to modulate force potentiation mechanisms [35], it may be that the twitch force potentiation deteriorates in menopausal women, but this modulation was not large enough to be yet detected between the perimenopausal stages in the present study.

There were no differences between groups in knee extension strength, vertical jumping height, or ankle dorsiflexion strength. Our results suggest that these functional measures are not yet sensitive for a decline in present perimenopausal stages. Nevertheless, plantar flexion strength was higher in EP group than LP group and may point toward early modulation in muscle function in menopause. it should be noted that the decrease in hormonal levels in menopause is a risk factor for sarcopenia. It seems that the late perimenopausal stage does not yet reveal evidence for a decrease in muscle strength and function, although direction toward it may be observed [54].

This study has several limitations. Our sample size is relatively small, and the observed interactions should therefore be investigated in larger samples. TMS stimulation was performed without advanced navigation and information on individual brain images. Leg motor areas are relatively small, located deep in the inter-hemispheric fissure and the optimal stimulation points are less segregated than those of hand muscles [55]. Therefore, it can be challenging to find the optimal stimulation point and higher intensities may be required to elicit measurable MEP compared to hand [56]. For this, we used a stimulus intensity that was the individually detected lower limb RMT for each participant. This may provide a challenge for generalization, as different levels of stimulation intensities have resulted in different results in research. E.g. changes in SP after fatiguing exercise have been detected at lower TMS intensities but not at higher intensities [57]. It might be that the changes detected here are present at low stimulus intensities but not with higher ones. However, this requires further investigation. As the detection of RMT and SP may be challenging for the lower limb and older participants, we controlled our results for the intensity of the stimulator output. Furthermore, the number of TMS trials to achieve each MEP and SP average was rather small [12, 58]. Unlike the study by Swanson & Fling [50], we performed the stimulation only to the left hemisphere, where they did not find significant differences between young and older adults. Therefore, we cannot make further conclusions about hemispheric differences. Thus, our preliminary results require further investigation.

Our method to measure very low E2 levels with IMMULITE 2000 XPi has some inaccuracy, and thus the most subtle fluctuations may not be revealed. Therefore, all associations may not have become apparent. Our results of E2 levels were in line with FSH levels and menstrual histories. Our study participants did not include pre- or postmenopausal women; thus, we cannot draw inferences of how the detected modulation in inhibitory or excitatory processes relates to either the fertility stage or the postmenopausal stage.

## Conclusions

Our preliminary results indicate subtle modulation toward decreasing TMS-induced inhibition in the central nervous system and possibly decreasing muscle twitch force potentiation in perimenopausal women. Faultless interaction of inhibitory and excitatory processes is essential in appropriate motor control and our results suggest that the reduction of estrogens in menopausal transition may accelerate the aging process related to this interaction.

## Data Availability

The datasets used and analyzed during the current study are available from the corresponding author on reasonable request.

## Abbreviations

ANOVA: One-way analysis of variance
ANCOVA: Analysis of covariance
BMI: Body mass index
E2: 17β-Estradiol
EMG: Electromyography
EP: Early perimenopausal
FSH: Follicle-stimulating hormone
GABA: Gamma-aminobutyric acid
LP: Late perimenopausal
MEP: Motor evoked potential
MG: Medial gastrocnemius
MVC: Maximal voluntary contraction
Nm: Newton meters
pRLC: Phosphorylation of myosin regulatory light chains
PA: Physical activity
PTT: Peak twitch torque
RMT: Resting motor threshold
SD: Standard deviation
SP: Silent period
TA: Tibialis anterior
TMS: Transcranial magnetic stimulation
